# Census Bulletins 13, 14, and 15

**Published:** 1891-01

**Authors:** 


Census Bulletins, Nos. 13, 14, and 15. Hon. Robert P. Porter, Superintendent of Census.

No. 13 contains the statistics of steel, from which it appears that the output of steel for the year ending June 30th, 1890, was 4,466,926 tons, an increase over the year 1880 of 290 per cent.

No. 14 relates to the financial condition of 858 municipalities in the United States.

No. 15 relates to the census of Alaska, which is not yet completed, owing to the wild character of the country. W. M. J.

